# Supplementary material for: Predictors of Immunogenic Response to the BNT162b2 mRNA COVID-19 Vaccination in Patients with Autoimmune Inflammatory Rheumatic Diseases Treated with Rituximab
Source: Vaccines (Basel). 2022 Jun 6;10(6):901. doi: 10.3390/vaccines10060901 (PMC9229869; doi:10.3390/vaccines10060901)
Supplement: Supplementary file 1 [file vaccines-10-00901-s001.zip › vaccines-1707934-supplementary.pdf]

Table S1. Comparison of S1/S2-antibody levels and rituximab relevant variables in rituximab treated AIIRD patients categorized by disease.

| Variable | RA | SLE | AAV | IIM | Other vasculitides |
|---|---|---|---|---|---|
| Anti-S1/S2 antibody test level, BAU, mean±SD | 55.19±81.55 | 99.84±110.55 | 36.25±73 | 25.19±45.07 | 48.8±74.29 |
| Positive serology, n (%) | 23 (46.94) | 9 (81.82) | 6 (26.09) | 4 (22.22) | 2 (33.33) |
| Serum IgG level (prior to last RTX course), mg/dL median (range) ** | 987 (377-2428) | 1529 (700-2090) | 839 (357-1630) | 870 (457-3405) | 510.25 (368-2153) |
| RTX cumulative dose (mg) median (range) | 10,000 (1600-30,000) | 3250 (2000-18,000) | 4000 (1000-18,000) | 6000 (2000-13,000) | 13,750 (2000-20,000) |
| RTX dose of last course prior to vaccination, mg, median (range) | 2000 (1000-3210) | 2000 (500-2000) | 1000 (500-2000) | 2000 (500-2000) | 2000 (500-2000) |
| Total number of RTX courses, median (range) | 6 (1-15) | 2 (1-11) | 4 (1-13) | 3 (1-8) | 7.5 (1-12) |
| Time interval between last RTX course and vaccination, days, mean±SD; median (range) | 263.35±385.47 160 (10-2561) | 351.55±367.58 137 (2-1147) | 394.7±623.08 175 (36-2794) | 192.61±159.37 149.5 (44-667) | 192.5±74.41 165 (122-333) |

Legend: AIIRD, autoimmune inflammatory rheumatic diseases; AAV, antineutrophil cytoplasmic antibody associated vasculitis; BAU, binding antibody units; csDMARDS, conventional synthetic disease modifying antirheumatic drugs; IIM, idiopathic inflammatory myopathy; IgG, immune globulin G; IVIG, intravenous immune globulin; n, number; RA, rheumatoid arthritis; SD, standard deviation, RTX, rituximab; SLE, systemic lupus erythematosus. **： Data were missing for 3 patients.

Table S2. Demographic and clinical characteristics of the RTX-AIIRD patients in the validation group compared to the original study group.

| Variable | Study group N=108 | Validation group N=48 | p value |
|---|---|---|---|
| Positive SARS-CoV-2 IgG test result, n (%) | 45 (41.67) | 21 (42.31) | 0.861 |
| Age, years, mean±SD | 61.45±14.96 | 60.54±12.29 | 0.712 |
| Female, n (%) | 83 (76.85) | 39 (81.25) | 0.675 |
| *AIIRD diagnosis, n (%)* | | | |
| RA | 49 (45.37) | 32 (66.67) | 0.047 |
| PsA | 1 (0.93) | 0 (0) | |
| SLE | 11 (10.19) | 2 (4.17) | |
| AAV | 23 (21.3) | 3 (6.25) | |
| Other vasculitides | 6 (5.56) | 1 (2.08) | |
| IIM | 18 (16.67) | 10 (20.83) | |
| *Rituximab relevant details*, mean±SD | | | |
| Serum IgG level (prior last RTX course), mg/dL | 1009.42±458.80 | 1013.27±240.65 | 0.946 |
| Total number of RTX courses, mean±SD | 5.13±3.72 | 5.73±3.49 | 0.345 |
| Time interval between last RTX course and BNT162b2 vaccine, days, mean±SD | 290.31±414.58 | 301.71±302.2 | 0.847 |

Legend: AIIRD, autoimmune inflammatory rheumatic diseases; AAV, antineutrophil cytoplasmic antibody associated vasculitis; idiopathic inflammatory myopathy; IgG, immune globulin G; PsA, psoriatic arthritis; RA, rheumatoid arthritis; SD, standard deviation; RTX, rituximab; SLE, systemic lupus erythematosus.

Table S3. Adverse reactions to the BNT162b2 mRNA vaccine in rituximab treated AIIRD patients treated categorized by the immunogenic response to vaccination.

| | Patients with a positive immunogenic response, n=45 | | Patients with a negative immunogenic response, n=61 | | p value | |
|---|---|---|---|---|---|---|
| Timing after vaccine doses | 1st dose | 2nd dose | 1st dose | 2nd dose | 1st dose | 2nd dose |
| **Local reactions, n (%)** | | | | | | |
| Pain | 22 (48.89) | 22 (51.16) | 33 (54.1) | 27 (44.26) | 0.695 | 0.552 |
| Erythema | 1 (2.22) | 3 (6.98) | 2 (3.28) | 2 (3.28) | 1 | 0.647 |
| Swelling | 4 (9.09) | 3 (6.98) | 0 (0) | 1 (1.64) | 0.026 | 0.304 |
| Pruritus | 1 (2.27) | 1 (2.33) | 1 (1.67) | 0 (0) | 1 | 0.414 |
| Tingling | 0 (0) | 0 (0) | 0 (0) | 0 (0) | 1 | 1 |
| **Systemic reactions, n (%)** | | | | | | |
| Fever>38°C | 0 (0) | 3 (7.14) | 1 (1.67) | 5 (8.2) | 1 | 1 |
| Nausea | 1 (2.27) | 2 (4.65) | 1 (1.67) | 2 (3.28) | 1 | 1 |
| Vomiting | 0 (0) | 0 (0) | 0 (0) | 0 (0) | 1 | 1 |
| Rhinorrhea | 0 (0) | 0 (0) | 0 (0) | 0 (0) | 1 | 1 |
| Cough | 1 (2.27) | 0 (0) | 0 (0) | 0 (0) | 0.423 | 1 |
| Myalgia | 0 (0) | 1 (2.33) | 1 (1.67) | 5 (8.2) | 1 | 0.397 |
| Arthralgia | 1 (2.27) | 1 (2.33) | 0 (0) | 2 (3.28) | 0.423 | 1 |
| Chills | 0 (0) | 7 (16.35) | 1 (1.67) | 10 (16.39) | 1 | 1 |
| Malaise | 2 (4.55) | 3 (6.98) | 1 (1.67) | 4 (6.56) | 0.572 | 1 |
| Headache | 4 (9.09) | 8 (18.6) | 3 (5) | 6 (9.84) | 0.453 | 0.248 |
| Allergic reaction | 0 (0) | 0 (0) | 0 (0) | 0 (0) | 1 | 1 |
| Lethargy | 3 (6.82) | 8 (18.6) | 1 (1.67) | 8 (13.11) | 0.308 | 0.582 |
| Worsening of rheumatologic symptoms | 1 (2.27) | 1 (2.33) | 1 (1.67) | 1 (1.64) | 1 | 1 |
| **Other adverse reactions, n** | Palpitations 1<br>Pruritus 1<br>Weakness 2 | Weakness and high BP 1<br>Weakness 2<br>Vaginal bleeding 1<br>Pericarditis 1 | Palpitations 1<br>Dizziness 2 | Dizziness 1<br>Nausea 1<br>Death 1 | | |

| | | | | | | |
|---|---|---|---|---|---|---|
| | Throat pain 1<br>Night sweats 1<br>Feeling heavy 1 | Dizziness 2<br>Sweating 2<br>Ear pain 1 | | | | |

Legend: AIIRD, autoimmune inflammatory rheumatic disease; BP, blood pressure; n, number.

Table S4. Disease activity scores pre- and post-BNT162b2 mRNA vaccination in in rituximab treated AIIRD patients treated categorized by the immunogenic response to vaccination.

| Disease activity score | Pre/post vaccine | All AIIRD patients (by diagnoses) | Vaccine responders | Vaccine non-responders | p value |
|---|---|---|---|---|---|
| RA-CDAI,<br>(n) median (range) | Pre | (42) 7 (0-40) | (21) 11 (0-35) | (21) 6 (0-40) | 0.536 |
| | Post | (41) 11 (0-41) | (20) 11.5 (0-41) | (21) 9 (0-27) | 0.327 |
| | Change | (39) 0 (-17-22) | (19) 1 (-8.5-22) | (20) 0 (-17-16) | 0.146 |
| RA-SDAI,<br>(n) median (range) | Pre | (37) 6.5 (0-40.65) | (17) 6 (0-35.3) | (20) 6.75 (0.06-40.65) | 0.842 |
| | Post | (35) 11 (0-41.5) | (17) 15 (0-41.5) | (18) 9.83 (1-27) | 0.33 |
| | Change | (34) 0.32 (-17.18-22) | (16) 2 (-8.3-22) | (18) 0.15 (-17.18-15.94) | 0.117 |
| RA-DAS28-CRP,<br>(n) median (range) | Pre | (37) 2.6 (0.97-5.86) | (17) 2.44 (0.97-5.2) | (20) 2.63 (1.13-5.86) | 0.773 |
| | Post | (35) 3.03 (1.1-5.92) | (17) 3.56 (1.1-5.92) | (18) 2.88 (1.24-4.98) | 0.485 |
| | Change | (34) 0.06 (-1.87-2.65) | (16) 0.35 (-1.27-2.65) | (18) 0.03 (-1.87-1.9) | 0.188 |
| SLE-SLEDAI,<br>(n) median (range) | Pre | (10) 2.5 (0-6) | (8) 2.5 (0-6) | (2) 3 (2-4) | 0.767 |

| | | | | | |
|---|---|---|---|---|---|
| | Post | (9) 4 (0-6) | (7) 4 (0-6) | (2) 3 (2-4) | 1 |
| | Change | (9) 0 (0-4) | (7) 0 (0-4) | (2) 0 (0-0) | 0.356 |
| AAV-PGA, (n) median (range) | Pre | (21) 0 (0-5) | (5) 1 (0-4) | (16) 0 (0-5) | 0.264 |
| | Post | (20) 0 (0-10) | (4) 0 (0-1) | (16) 0 (0-10) | **0.05** |
| | Change | (20) 0 (-4-10) | (4) 0 (-4-0) | (16) 0 (-1-10) | 0.18 |
| AAV-PhGA, (n) median (range) | Pre | (21) 0 (0-6) | (4) 0 (0-1) | (17) 0 (0-6) | 0.243 |
| | Post | (20) 0 (0-10) | (4) 0 (0-1) | (16) 0 (0-10) | 0.136 |
| | Change | (20) 0 (0-10) | (4) 0 (0-0) | (16) 0 (0-10) | 0.215 |
| IIM-PGA, (n) median (range) | Pre | (14) 2 (0-9) | (3) 1.5 (0-7) | (11) 2 (0-9) | 0.869 |
| | Post | (14) 2 (0-9) | (3) 1.5 (0-7) | (11) 2 (0-9) | 0.993 |
| | Change | (14) 0 (0-2) | (3) 0 (0-0) | (11) 0 (0-2) | 0.192 |
| IIM-PhGA, (n) median (range) | Pre | (14) 2 (0-5) | (3) 2 (0-4) | (11) 2 (0-5) | 0.964 |
| | Post | (14) 2 (0-5) | (3) 2 (0-4) | (11) 2 (0-5) | 0.967 |
| | Change | (14) 0 (-2-2) | (3) 0 (0-0) | (11) 0 (-2-2) | 1 |
| Other vasculitis-PGA, (n) median (range) | Pre | (6) 0 (0-5) | (2) 2.5 (0-5) | (4) 0 (0-4) | 0.523 |
| | Post | (5) 0 (0-5) | (2) 2.5 (0-5) | (3) 0 (0-0) | 0.5 |
| | Change | (5) 0 (0-0) | (2) 0 (0-0) | (3) 0 (0-0) | NA |
| Other vasculitis-PhGA, (n) median (range) | Pre | (5) 0 (0-1) | (2) 0.5 (0-1) | (3) 0 (0-0) | 0.5 |
| | Post | (5) 0 (0-1) | (2) 0.5 (0-1) | (3) 0 (0-0) | 0.5 |
| | Change | (5) 0 (0-0) | (2) 0 (0-0) | (3) 0 (0-0) | NA |

Legend: AIIRD, autoimmune inflammatory rheumatic diseases; AAV, antineutrophil cytoplasmic antibody associated vasculitis; CDAI, clinical disease activity index; DAS28-CRP, disease activity score-

28-CRP; n, number; PGA, patient global assessment; PhGA, physician global assessment; RA, rheumatoid arthritis; SD, standard deviation; SDAI, simplified disease activity index; SLE, systemic lupus erythematosus; SLEDAI, systemic lupus erythematosus disease activity index

Supplementary Figure S1. Changes in disease activity scores for rheumatoid arthritis patients treated with rituximab according to immunogenic response to the BNT162b2 mRNA vaccine.

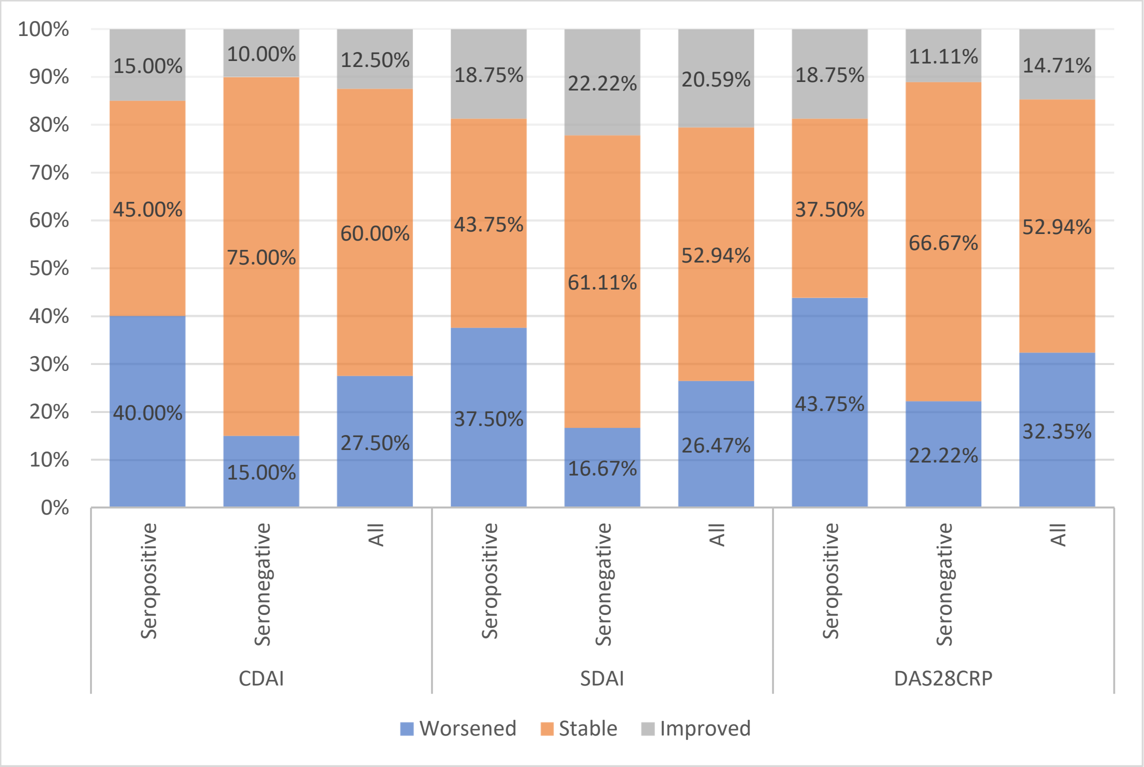


Data were available before and after vaccination for 40 rheumatoid arthritis patients for CDAI, 34 for SDAI, and 34 DAS-28-CRP. Differences between patients who mounted a seropositive immunogenic response to vaccination and those who did not were non-significant (p values 0.177, 0.43, and 0.234, respectively).

Legend: CDAI, clinical disease activity index; DAS28-CRP, disease activity score-28-CRP;

SDAI, simplified disease activity index.
